# COVID-19 Outbreak Among Three Affiliated Homeless Service Sites — King County, Washington, 2020

**DOI:** 10.15585/mmwr.mm6917e2

**Published:** 2020-05-01

**Authors:** Farrell A. Tobolowsky, Elysia Gonzales, Julie L. Self, Carol Y. Rao, Ryan Keating, Grace E. Marx, Temet M. McMichael, Margaret D. Lukoff, Jeffrey S. Duchin, Karin Huster, Jody Rauch, Hedda McLendon, Matthew Hanson, Dave Nichols, Sargis Pogosjans, Meaghan Fagalde, Jennifer Lenahan, Emily Maier, Holly Whitney, Nancy Sugg, Helen Chu, Julia Rogers, Emily Mosites, Meagan Kay

**Affiliations:** ^1^Epidemic Intelligence Service, CDC; ^2^CDC COVID-19 Emergency Response; ^3^Public Health – Seattle & King County, Washington; ^4^University of Washington, Seattle, Washington; ^5^King County Department of Community and Human Services, Seattle, Washington.

On March 30, 2020, Public Health – Seattle and King County (PHSKC) was notified of a confirmed case of coronavirus disease 2019 (COVID-19) in a resident of a homeless shelter and day center (shelter A). Residents from two other homeless shelters (B and C) used shelter A’s day center services. Testing for SARS-CoV-2, the virus that causes COVID-19, was offered to available residents and staff members at the three shelters during March 30–April 1, 2020. Among the 181 persons tested, 19 (10.5%) had positive test results (15 residents and four staff members). On April 1, PHSKC and CDC collaborated to conduct site assessments and symptom screening, isolate ill residents and staff members, reinforce infection prevention and control practices, provide face masks, and advise on sheltering-in-place. Repeat testing was offered April 7–8 to all residents and staff members who were not tested initially or who had negative test results. Among the 118 persons tested in the second round of testing, 18 (15.3%) had positive test results (16 residents and two staff members). In addition to the 31 residents and six staff members identified through testing at the shelters, two additional cases in residents were identified during separate symptom screening events, and four were identified after two residents and two staff members independently sought health care. In total, COVID-19 was diagnosed in 35 of 195 (18%) residents and eight of 38 (21%) staff members who received testing at the shelter or were evaluated elsewhere. COVID-19 can spread quickly in homeless shelters; rapid interventions including testing and isolation to identify cases and minimize transmission are necessary. CDC recommends that homeless service providers implement appropriate infection control practices, apply physical distancing measures including ensuring resident’s heads are at least 6 feet (2 meters) apart while sleeping, and promote use of cloth face coverings among all residents (*1*).

The first COVID-19 case in the United States was confirmed in Snohomish County, Washington, on January 20, 2020. The governor of Washington issued stay-at-home orders on March 23; by March 28, a total of 2,307 confirmed COVID-19 cases had been reported in nearby King County (*2*,*3*). On March 30, PHSKC was notified that a resident of homeless shelter A had positive test results for SARS-CoV-2 (Figure). The resident, a man aged 67 years with underlying medical conditions, was hospitalized on March 29 for acute encephalopathy. He reported 2 days of cough, shortness of breath, fever, sore throat, and runny nose. A nasopharyngeal swab collected on admission was positive for SARS-CoV-2 by real-time reverse transcription–polymerase chain reaction testing. The patient remained clinically stable without the need for intensive care unit support and was discharged after 5 days to isolation housing (i.e., an individual room with clinical support) provided by the King County Department of Community and Human Services.

**FIGURE:**
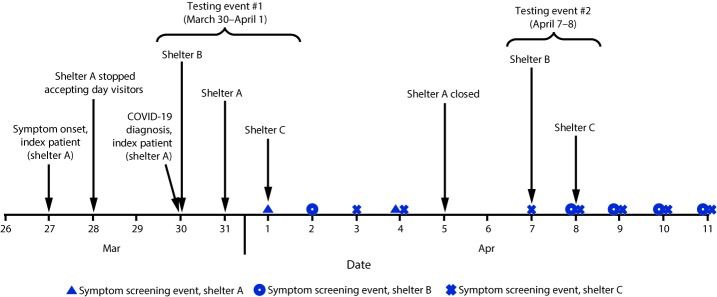
Testing events and changes in practices in response to a COVID-19 outbreak at three affiliated homeless shelters — King County, Washington, March 27–April 11, 2020 **Abbreviation:** COVID-19 = coronavirus disease 2019.

During March 30–April 1, SARS-CoV-2 testing was offered to all available residents and staff members at shelter A, as well as those at shelters B and C, which used shelter A’s day services (testing event 1). Overall, 62.8% of residents who spent the previous night at each shelter were tested. Residents and staff members were not screened for symptoms before testing. At shelter A, seven of 43 residents and four of 15 staff members had positive test results (Table 1). Two of 74 residents at shelter B and six of 37 residents at shelter C had positive test results. None of the staff members tested from shelters B and C had positive test results. Twelve residents with confirmed SARS-CoV-2 infection identified by testing event 1 were transported to isolation housing, and three were hospitalized; staff members with confirmed infection self-isolated at home.

**TABLE 1:** Number of residents and staff members tested for SARS-CoV-2 and number and percentage who had positive test results at two testing events — three affiliated shelters, Seattle, Washington, March 30–April 8, 2020

Shelter	Testing event 1 (March 30–April 1, 2020)					Testing event 2 (April 7–8, 2020)*				
	Residents			Staff members		Residents			Staff members	
	No. eligible^†^	No. tested	No. (%) positive	No. tested^§^	No. (%) positive	No. eligible*	No. tested	No. (%) positive	No. tested	No. (%) positive
Shelter A	43	43	7 (16.3)	15	4 (26.7)	7^¶^	7	2 (28.6)	N/A**	N/A**
Shelter B	109	74	2 (2.7)	2	0 (—)	87	52	4 (7.7)	8	1 (12.5)
Shelter C	93	37	6 (16.2)	10	0 (—)	79	44	10 (22.7)	7	1 (14.3)
**Total**	**245**	**154**	**15 (9.7)**	**27**	**4 (14.8)**	**173**	**103**	**16 (15.5)**	**15**	**2 (13.3)**

**Abbreviation:** N/A = not applicable.

* Residents and staff members who had negative test results or were not available in testing event 1 were tested in event 2.

^†^ Residents were eligible for testing if they spent the previous night at the shelter.

^§^ Total number of staff members working the day of testing was not available.

^¶^ Female residents from shelter A who were tested at isolation housing after shelter A closed on April 5, 2020.

** Shelter closed.

A CDC team arrived April 1 to support PHSKC rapid response teams. The teams assessed 122 residents and staff members over 3 days to identify COVID-19–like illness (i.e., new or worsening cough, dyspnea, or subjective or measured fever [temperature ≥100.4°F (38°C)]), conducted site assessments at each shelter, and provided recommendations to limit transmission at the three shelters.

Shelter A is a 24-hour shelter that served up to 40 men and 10 women; sleeping mats (not assigned to individual residents) were arranged in two rooms during the night and stacked during the day. Shelter B housed up to 110 men in two main rooms; shelter C housed up to 100 men in two main rooms. To reduce crowding and COVID-19 transmission risk, approximately half of the residents of shelter B had been transferred to shelter C on March 13. Sleeping mats and locations in shelters B and C were assigned to individual residents and remained in place all day. Shelters B and C became 24-hour shelters on March 13 and 26, respectively. All shelters had onsite indoor bathrooms with sinks and soap. All shelters served persons aged ≥50 years and were located approximately 2–5 miles (3–8 kilometers) from each other.

Site assessments identified multiple areas for improvement in sheltering-in-place and infection prevention and control practices. Staff members rotated among the three shelters. Residents were able to leave the shelters if they returned by curfew. Sleeping mats in each of the shelters were spaced ≤3 feet apart. Shelter C did not have alcohol-based hand sanitizer or on-site showers; residents used shelter shuttles or public transportation to access public showers. Staff members intermittently wore cloth face coverings or face masks; however, these were not provided to residents.

Following the assessment, recommendations to decrease the risk for COVID-19 transmission were implemented. On April 5, to address staffing shortages, PHSKC recommended closing shelter A and relocating women residents of shelter A to isolation housing with individual rooms and relocating men to shelter C, where PHSKC provided thermometers for temperature screening and arranged for portable showers to prevent the need for public shower facility use (Figure). For all shelters, the rapid response teams provided recommendations to limit staff member rotations, encourage physical distancing, limit movement in and out of the shelter, train staff members on cleaning and disinfection, and move sleeping mats so that residents’ heads are ≥6 feet (≥2 meters) apart. Disposable face masks were provided to all residents and staff to aid in source control.

PHSKC coordinated active case finding and during April 7–8 conducted repeat SARS-CoV-2 testing (testing event 2) of all available residents and staff members who had negative test results or were unavailable for the first testing. This testing event identified additional confirmed COVID-19 cases among 16 of 103 (15.5%) residents and two of 15 (13.3%) staff members (Table 1). During April 1–11, PHSKC also conducted 14 symptom screening events among residents and staff members across all three shelters. Persons with COVID-19–like illness were connected to testing, which identified two additional cases among residents. Two staff members and two residents each sought health care independently and had positive test results for SARS-CoV-2.

By April 11, 2020, testing confirmed COVID-19 among 35 residents and eight staff members. Among these 43 confirmed cases, 37 (86%) were identified through testing offered to everyone at the shelter, two (5%) through symptom screening, and four (9%) after persons independently sought health care (Table 2). Among residents with confirmed COVID-19, the median age was 61 years (range = 50–73 years) and among staff members was 39 years (range = 28–57 years). Overall, 187 of 195 (96%) residents tested were men; among residents who had positive test results for COVID-19, 31 (89%) were men. Seven residents (20%) were hospitalized; none has died to date. No staff members were hospitalized or died.

**TABLE 2:** Number and percentage of shelter residents and staff members with COVID-19 diagnosed by testing, symptom screening, or independent health care evaluation — Seattle, Washington, March 30–April 11, 2020

Method of diagnosis	No. (%) with COVID-19 diagnosis	
	Residents assessed (N = 195)	Staff members assessed (N = 38)
Testing event 1	15 (8)	4 (11)
Testing event 2	16 (8)	2 (5)
Symptom screening	2 (1)	—
Evaluated elsewhere	2 (1)	2 (5)
**Total**	**35 (18)**	**8 (21)**

**Abbreviation:** COVID-19 = coronavirus disease 2019.

## Discussion

This COVID-19 outbreak involved transmission among residents and staff members of three affiliated homeless shelters in Seattle, Washington. Conditions that might have contributed to SARS-CoV-2 transmission in these sites include 1) the mobile nature of the community and use of multiple homeless service sites among residents; 2) crowding and use of congregate sleeping arrangements; 3) challenges enforcing physical distancing; 4) possible asymptomatic transmission; and 5) unavailability of face coverings for residents before public health intervention.

PHSKC, the King County Department of Community and Human Services, and homeless service site leadership implemented rapid public health interventions to minimize transmission by proactively testing all residents and staff members and promptly transporting symptomatic and residents with confirmed disease to isolation housing. Additional measures included limiting movement into and out of the shelter (e.g., by providing on-site showers), encouraging physical distancing, and making infection prevention and control recommendations. Response coordination required resource investment and collaboration between local health and community service departments, staff members at homeless shelter sites, community health care providers, and federal partners.

The findings in this report are subject to at least four limitations. First, not all residents were present during the site visits; thus, residents with SARS-CoV-2 infection could have been missed during the testing events or symptom screening. Multiple testing and screening events were conducted to assess as many residents as possible. Second, these public health interventions were resource-intensive, which might not be sustainable long term. Third, symptom screening and testing were conducted independently of each other, which did not allow for simple linkage of symptom and test result information. Finally, the effectiveness of the interventions could not be assessed during the period of the investigation and response.

Homeless service sites are densely populated environments, similar to long-term care facilities, which can amplify infectious disease outbreaks, including COVID-19 (*4*). Common methods to control COVID-19 spread (e.g., testing, contact tracing, physical distancing, and restricting movement) are difficult to implement among persons who are experiencing homelessness (*5*,*6*), and stay-at-home orders are impractical. CDC has published interim guidance for homeless service providers to plan and respond to COVID-19. CDC recommends that homeless service providers implement appropriate infection control practices, apply physical distancing measures including ensuring resident’s heads are at least 6 feet apart while sleeping, and promote use of cloth face coverings among all residents (*1*). Assistance with enforcement of shelter-in-place orders might be necessary for persons experiencing homelessness during spread of COVID-19. At shelters experiencing COVID-19 outbreaks, transferring infected residents and those with underlying health conditions or of advanced age (*7*,*8*) into individual housing units should be prioritized.

In this outbreak, testing events for everyone in the shelter identified a high proportion (86%) of COVID-19 cases and allowed for prompt transfer to isolation housing. Evidence exists for presymptomatic and asymptomatic transmission of SARS-CoV-2 (*9*); the testing of all available residents and staff members regardless of symptoms performed during this investigation potentially identified more infectious cases than symptomatic screening would have. Prompt implementation of public health interventions to identify COVID-19 cases early can mitigate further transmission in jurisdictions at high risk for community transmission.

**Summary**

**What is already known about this topic?**

COVID-19 can spread rapidly within and between congregate housing facilities, such as homeless shelters. COVID-19 in homeless shelters, however, has not been well described.

**What is added by this report?**

On April 1, 2020, a COVID-19 outbreak was detected at three affiliated homeless shelters. Testing for SARS-CoV-2 immediately offered to all residents and staff members identified additional unrecognized COVID-19 cases. Enhanced surveillance and repeat testing identified and confirmed COVID-19 in 43 persons at these sites.

**What are the implications for public health practice?**

Interrupting COVID-19 transmission in homeless shelters is challenging. In settings with known COVID-19 outbreaks, assistance with enforcement of shelter-in-place orders, testing of residents and staff members, and prompt isolation of symptomatic or residents with confirmed disease are needed to prevent further transmission in homeless shelters.
